# What Is the Price of Science?

**DOI:** 10.1128/mBio.00117-21

**Published:** 2021-03-02

**Authors:** James C. Alwine, Lynn W. Enquist, Terence S. Dermody, Felicia Goodrum

**Affiliations:** a Department of Cancer Biology, University of Pennsylvania School of Medicine, Philadelphia, Pennsylvania, USA; b Department of Immunobiology, University of Arizona, Tucson, Arizona, USA; c Department of Molecular Biology, Princeton University, Princeton, New Jersey, USA; d Department of Pediatrics, University of Pittsburgh School of Medicine, Pittsburgh, Pennsylvania, USA; e Department of Microbiology and Molecular Genetics, University of Pittsburgh School of Medicine, Pittsburgh, Pennsylvania, USA; f Institute of Infection, Inflammation, and Immunity, University of Pittsburgh Medical Center Children’s Hospital of Pittsburgh, Pittsburgh, Pennsylvania, USA; g BIO5 Institute, University of Arizona, Tucson, Arizona, USA; Columbia University/HHMI

**Keywords:** for-profit journals, impact factor, open access, preprint servers

## Abstract

The peer-reviewed scientific literature is the bedrock of science. However, scientific publishing is undergoing dramatic changes, which include the expansion of open access, an increased number of for-profit publication houses, and ready availability of preprint manuscripts that have not been peer reviewed. In this opinion article, we discuss the inequities and concerns that these changes have wrought.

## OBSERVATION

Scientific literature is a roadmap for the worldwide scientific endeavor. It is how scientists build on the findings of other scientists, even those from earlier times. The publication of rigorously vetted results and conclusions is how we mark scientific advancement.

Peer review is the foundation of scientific publication. When it operates properly, peer review verifies that the conclusions are justified by the results shown and assesses the overall novelty and significance of the work. At its best, peer review stands as one of the few barriers between meaningful science and weak, misleading, or inaccurate reports.

We are now in the midst of a sea change in scientific publishing, which unveils troubling inequities.

Historically, scientific manuscripts were published in journals that lined library and office shelves. Nonprofit scientific societies provided the services of publication using dues and subscription fees (personal and institutional) to cover much of the costs. Editors and peer reviewers were provided minimal, if any, financial support. The scientific community considered these roles essential responsibilities to the scientific endeavor and served them voluntarily, a tradition that remains to this day. Any excess funds realized from scientific publishing were contributed back to the sponsoring organization to support scientific meetings and professional activities. As a result of this business model, publication fees charged to authors (e.g., for page and figure charges) were designed only to cover costs.

However, in recent years, there has been a major shift to electronic publication with the promotion of “open access,” in which publications are freely available to all. The motivation is clear. Science is truly the public’s product, and the public should have access to science without delay. However, despite the egalitarian aspects of open access, its business model is challenging. How can expenses be covered when the product is free?

This question has been answered by the appearance of many new “for-profit” publication houses that use paid professional editors: make the authors pay. These entities are subsuming the traditional role of scientific societies. For these publishers, profit is the primary motive, sometimes at the expense of scientific quality or integrity. The reader is no longer the significant payer. Instead, authors are increasingly responsible for bearing the cost of open-access publishing.

Some publishers (1), such as Springer Nature (2), charge large fees to authors for open-access publication. For example, the fee to publish an open-access paper in *Nature* is approximately $11,000. Springer Nature also proposes charging the authors a fee of approximately $2,000 for reviewing a manuscript for Nature, which, given the journal, has a strong probability of being rejected even when the premise and rigor of the study is strong.

Increasing profits by publishing houses has been a growing problem for years. As early as 2010, “Elsevier’s scientific publishing arm reported profits of £724 million on just over £2 billion in revenue. That is a 36% profit margin—higher than Apple, Google, or Amazon posted that year” (3). This profit margin is being generated under conditions where authors generate the “product” (no cost to the publisher) and pay open-access fees, reviewers peer review the paper for free (no cost to the journal), and readers/institutions pay to access non-open-access articles.

In contrast, the nonprofit American Society for Microbiology (ASM) has seven “hybrid” journals, and each supports open access. ASM members are charged $90 per page for a non-open-access publication in one of the hybrid journals. Notably, free open access for such papers is provided after 6 months. If authors choose immediate open access, the charge is $2,500. There is no charge to review papers submitted to ASM journals.

The more egregious problem posed by the for-profit business model is that it subverts science by engendering inequity. Scientists usually pay publication costs from grants intended to support research. Therefore, only highly funded scientists or those at elite institutions that cover publication costs will clear this new barrier to publication. More modestly funded investigators, often younger scientists, those at smaller colleges or universities, or those working in more resource-limited settings, will be in effect prohibited from publishing in such high-profile journals simply because it costs too much.

Another troubling change concerns the increasing importance of impact factor in choosing a venue to publish or judging the quality of a paper. Impact factor reflects the yearly average number of citations for articles published in a given journal in the previous 2 years. It is often used as a proxy for the relative standing of a journal. Judgments about promotion, tenure, and field leadership are often, to the detriment of science, centered on the impact factor of the journals in which a scientist publishes. The inappropriate use of impact factor as a symbol of quality and prestige has much to do with the inequities in publication and the decline of submissions to society journals.

The consequence of charging authors substantial fees for reviewing papers and open-access publication in prestigious journals is that it will slowly select against smaller labs and concentrate scientific funding and influence in the most well-supported labs, perhaps only the top 5 to 10%, leaving the rest to struggle.

This path is not sustainable. Science has long benefited from rigorous experimentalists at all levels of academia and industry. The contributions of scientists working at smaller institutions or in fields that do not attract significant funding are as vital as contributions made by those employed at the wealthiest and most powerful institutions. That mission encompasses not only the science but extends to the training of the next generation of scientists. Science thrives when those from diverse backgrounds and perspectives are drawn to the endeavor. For publishers to benefit from large profit margins, while scientist struggle to keep labs open, is simply wrong.

There are more changes afoot with the appearance of several online open-access sites for free posting of preprints: no peer review, no page changes, and no fees. Their appearance questions the purpose of journals. Does acceptance by a journal simply place a very costly stamp of approval on an already “published” preprint? Why bother with peer review? The posted preprint can be viewed, read, referred to, and used by other scientists just as easily whether it is reviewed or not.

Financially beleaguered investigators may find it tempting to forgo the many benefits and improvements provided by peer review and simply post the preprint and circumvent expensive publication. Such action would sadly increase the number and availability of unvetted unsubstantiated reports, which is dangerous. The press and the public do not heed the difference between peer-reviewed publications and preprints. In this time of increasing misinformation, it is important that we rigorously assess and maintain quality and resist expediency or the false lure of impact.

It is surprising that more rank-and-file scientists have not expressed concerns with the ramifications of this upheaval in publication. There is a parable of a frog in the kettle of slowly heating water. Initially the frog is comfortable, then things get progressively worse, and unless the frog jumps out, the frog is cooked. The changes in scientific publication should make scientists very uncomfortable. It is time to jump out and push back.

What is the way forward? It is essential to maintain journalistic standards, integrity in scientific publishing, and access. The ability to publish meritorious science in any journal should not be deterred by cost. The perception that impact factor defines journal quality must be reconsidered. Society journals should be respected and supported as the bedrock of science. They offer a forum to evaluate science for its rigor and merit by other scientists, with editors who work with authors without undue regard for press appeal, all at an affordable price.
